# Polygenic risk scores enhance prediction of body mass index increase in individuals with a first episode of psychosis

**DOI:** 10.1192/j.eurpsy.2023.9

**Published:** 2023-02-28

**Authors:** Gerard Muntané, Javier Vázquez-Bourgon, Ester Sada, Lourdes Martorell, Sergi Papiol, Elena Bosch, Arcadi Navarro, Benedicto Crespo-Facorro, Elisabet Vilella

**Affiliations:** 1 Hospital Universitari Institut Pere Mata, IISPV, Universitat Rovira i Virgili, Reus, Spain; 2 Centro de Investigación Biomédica en Red de Salud Mental (CIBERSAM), Madrid, Spain; 3 Institut de Biologia Evolutiva (UPF-CSIC), Department of Medicine and Life Sciences, Universitat Pompeu Fabra, Parc de Recerca Biomèdica de Barcelona, Barcelona, Spain; 4 Department of Psychiatry, University Hospital Marqués de Valdecilla, Instituto de Investigación Marqués de Valdecilla (IDIVAL), Santander, Spain; 5 Departamento de Medicina y Psiquiatría, Facultad de Medicina, Universidad de Cantabria, Santander, Spain; 6 Department of Psychiatry, Institute of Psychiatric Phenomics and Genomics, University Hospital, Ludwig Maximilian University, Munich, Germany; 7 Centre for Genomic Regulation (CRG), The Barcelona Institute of Science and Technology, Barcelona, Spain; 8 Institució Catalana de Recerca i Estudis Avançats (ICREA), Barcelona, Spain; 9 Barcelonaβeta Brain Research Center, Fundació Pasqual Maragall, Barcelona, Spain; 10 Department of Psychiatry, Instituto de Biomedicina de Sevilla (IBiS), University Hospital Virgen del Rocío, Seville, Spain

**Keywords:** BMI, first-episode psychosis, pleiotropy, polygenic risk scores, weight gain

## Abstract

**Background:**

Individuals with a first episode of psychosis (FEP) show rapid weight gain during the first months of treatment, which is associated with a reduction in general physical health. Although genetics is assumed to be a significant contributor to weight gain, its exact role is unknown.

**Methods:**

We assembled a population-based FEP cohort of 381 individuals that was split into a Training (*n* = 224) set and a Validation (*n* = 157) set to calculate the polygenic risk score (PRS) in a two-step process. In parallel, we obtained reference genome-wide association studies for body mass index (BMI) and schizophrenia (SCZ) to examine the pleiotropic landscape between the two traits. BMI PRSs were added to linear models that included sociodemographic and clinical variables to predict BMI increase (∆BMI) in the Validation set.

**Results:**

The results confirmed considerable shared genetic susceptibility for the two traits involving 449 near-independent genomic loci. The inclusion of BMI PRSs significantly improved the prediction of ∆BMI at 12 months after the onset of antipsychotic treatment by 49.4% compared to a clinical model. In addition, we demonstrated that the PRS containing pleiotropic information between BMI and SCZ predicted ∆BMI better at 3 (12.2%) and 12 months (53.2%).

**Conclusions:**

We prove for the first time that genetic factors play a key role in determining ∆BMI during the FEP. This finding has important clinical implications for the early identification of individuals most vulnerable to weight gain and highlights the importance of examining genetic pleiotropy in the context of medically important comorbidities for predicting future outcomes.

## Introduction

Schizophrenia (SCZ) is a severe disorder with a large burden of morbidity and societal impact. It has a heritability of ~80%, much of which is attributable to common risk alleles [1]. Patients with SCZ often have a large number of comorbid medical conditions during their lifespan [2, 3]. In fact, people with SCZ have a higher mortality rate than the general population, corresponding to a 10–20 year reduction in life expectancy, predominantly due to cardiovascular disease [4, 5].

Antipsychotic (AP) drugs can help reduce the intensity and frequency of psychotic symptoms; however, most of them are obesogenic [6]. There are differences in the weight gain caused by AP drugs, with olanzapine and clozapine presenting a higher risk [7]. Obesity is a modifiable risk factor that reduces the quality of life [8], adherence to treatment [9], and is associated with many adverse health-related outcomes, including cardiovascular disease [10, 11]. The risk of obesity in patients with SCZ is more than four times higher than in the general population [12]. In fact, the first year after the initiation of AP treatment is a critical period in which up to 60–80% of the total weight gain occurs [13, 14]. Among all factors studied, rapid initial weight gain, AP drug, pretreatment body mass index (BMI), and sex are the best predictors of weight gain and associated metabolic abnormalities [15–17]. However, substantial differences in susceptibility between individuals under AP treatment suggest that weight gain may be partially explained by a mixture of environmental effects and genetic background [18–20]. In support of this view, the heritability of weight gain in monozygotic twins with SCZ has been estimated to be 0.6–0.8 [21] and a few genetic loci have been associated with AP-induced weight gain by genome-wide association studies (GWAS) [22, 23]. Moreover, given its strong polygenic component, SCZ shows extensive genetic overlap with other mental disorders [24, 25], and also with other nonpsychiatric traits [26–28]. In particular, studies have reported a negative overall genetic correlation between BMI and SCZ, which results from a mixture of variants with concordant and discordant effects [28–30].

There is a substantial genetic vulnerability to BMI trajectories in the general population [31, 32]; however, it remains to be established whether prediction models, including the polygenic risk score (PRS) for BMI (PRS_BMI_), are clinically useful for populations with psychiatric disorders. In this study, we aimed to determine whether common genetic variants for BMI confer a risk of BMI increase at treatment initiation in patients with first episode of psychosis (FEP), and to investigate the role of shared and private BMI variants in BMI increase (∆BMI). To our knowledge, this is the first study to confirm the role of genetics in FEP-associated weight gain, with a significant contribution of shared variants between the two traits, highlighting their relevance and applicability. This information can be used to identify individuals with an increased risk at the very early stages of the disease.

## Methods

### Study design

This study includes individuals with a FEP initially enrolled in the Cantabria Program for Early Intervention in Psychosis (PAFIP, Spain) between 2001 and 2018 [33]. Patients fulfilling inclusion criteria (Supplementary Material) were assigned to three consecutive phases of the PAFIP (PAFIP I, II, and III), including randomized, flexible-dose, and open-label clinical trials and followed up for 12 months. During this period, AP doses were adjusted at the treating psychiatrist’s discretion to target the lowest effective dose [34]. Similarly, AP treatment could be switched based on the observed effectiveness and the patient’s tolerance. For further analysis, the diagnosis was categorized into Schizophrenia, Schizoaffective disorder, Schizophreniform disorder, Brief psychotic disorder, and not otherwise specified psychosis. Written informed consent was obtained from all subjects. The Clinical Research Ethics Committee of Cantabria approved the research protocol.

Patients were evaluated for research purposes at three consecutive time points: at baseline, and at 3- and 12-month follow-ups (after the initiation of AP treatment). Sociodemographic characteristics were recorded at baseline (Table 1), while clinical and anthropometric measures were obtained at each time point. Pharmacological treatment prescribed and chlorpromazine equivalents [35] were also recorded at each time point. Patients were grouped based on the primary active drug (Table 2).

**Table 1.** Sociodemographic and clinical characteristics of the sample at baseline.

	Total (*n* = 381)	*Training set* (*n* = 224)	*Validation set* (*n* = 157)	Statistical difference
	*N* (%)	*N* (%)	*N* (%)	Pearson Chi-squared test
Sex (females)	165 (43.3)	98 (43.8)	67 (42.7)	*p* = 0.91*
Diabetes	7 (4.2)	7 (4.9)	0	*p* = 0.65*
Metabolic syndrome	14 (4.7)	11 (5.7)	3 (2.9)	*p* = 0.42*
Drug Naïve	354 (92.9)	202 (90.2)	152 (96.8)	*p* = 0.02*
Tobacco	211 (55.4)	121 (54)	90 (57.3)	*p* = 0.59*
Cannabis	153 (40.2)	85 (37.9)	68 (43.3)	*p* = 0.34*
Alcohol	192 (50.5)	101 (45.1)	91 (58.3)	*p* = 0.01*
Antidepressants	7 (1.8)	6 (2.7)	1 (0.6)	*p* = 0.28*
Stabilizers	1 (0.3)	1 (0.4)	0	*p* = 1*
*Diagnose after 6-months*				
Schizophrenia	188 (49.3)	96 (42.9)	92 (58.6)	*p* = 0.02
Schizophreniform disorder	115 (30.2)	73 (32.6)	42 (26.7)	
Brief psychotic disorder	43 (11.3)	32 (14.3)	11 (7)	
Unspecified psychosis	28 (7.3)	19 (8.5)	9 (5.7)	
Schizoaffective disorder	5 (1.3)	2 (0.9)	3 (2)	
Delusional disorder	2 (0.5)	2 (0.9)	0	

	Mean ± SD	Mean ± SD	Mean ± SD	Wilcoxon rank sum test
Age at admission, years	29.5 ± 9.3	30.7 ± 10.2	27.8 ± 7.6	*p* = 0.03
Weeks previous treatment	1.5 ± 1.6	1.4 ± 1.6	1.8 ± 1.7	*p* = 0.44
DUI, months	21.9 ± 37.2	16.7 ± 31.4	29 ± 43.1	*p* = 1.6e-04
DUP, months	13.3 ± 29.4	12.5 ± 31	14.4 ± 27	*p* = 0.04
HOMA baseline	2.2 ± 2.3	2.5 ± 2.7	1.7 ± 1.4	*p* = 1e-03
Weight	66.2 ± 13.6	65.6 ± 14	67.1 ± 13.1	*p* = 0.12
BMI	23.2 ± 3.8	23 ± 4	23.4 ± 3.5	*p* = 0.12
∆BMI_3_	6.4 ± 7.6	6 ± 7.3	7 ± 8	*p* = 0.21
∆BMI_12_	13.9 ± 12.1	13.6 ± 11.9	14.3 ± 12.4	*p* = 0.59

Abbreviations: DUI, duration of untreated illness; DUP, duration of untreated psychosis.

* Pearson’s Chi-squared test with Yates’ continuity correction.

**Table 2.** Number of participants in each treatment category at baseline and after 3 and 12 months of follow-up.

	Baseline		*t* = 3 months		*t* = 12 months	
Drug	Training	Validation	Training	Validation	Training	Validation
Aripiprazole	101 (45.1%)	11 (7%)	81 (38%)	9 (5.8%)	81 (38.6%)	9 (6.3%)
Clozapine	—	—	3 (1.4%)	—	10 (4.8%)	2 (1.4%)
Trifluoperazine	—	—	1 (0.5%)	—	—	—
Haloperidol	—	34 (21.7%)	—	31 (20.1%)	—	17 (11.9%)
Olanzapine	1 (0.4%)	42 (26.8%)	13 (6.1%)	45 (29.2%))	24 (11.4%)	46 (32.2%)
Paliperidone	—	—	3 (1.4%)	—	12 (5.7%)	—
Quetiapine	34 (21.7%)	15 (9.6%)	14 (6.6%)	12 (7.8%)	13 (6.2%)	18 (12.6%)
Risperidone	49 (21.9%)	43 (27.4%)	71 (33.3%)	49 (31.8%)	54 (25.7%)	44 (30.8%)
Ziprasidone	39 (24.8%)	12 (7.6%)	27 (12.7%)	8 (5.2%)	15 (7.1%)	7 (4.9%)
Amisulpride	—	—	—	—	1 (0.5%)	—
Total	224	157	213	154	210	143

### Samples and genotyping

For the present study, the PAFIP sample (*n* = 381) was divided into two independent patient datasets. Each of these datasets had been genotyped with a different platform. The first dataset (from now on *Training set*) included 224 patients and was used to determine the *p*-value threshold for PRSs, while a second sample (from now on *Validation set*) included 157 patients and was used for replication. In both datasets, DNA was extracted from peripheral lymphocytes, and genotyping was performed using the Affymetrix 6.0 platform (*Training set*) and the Illumina Infinium PsychArray (*Validation set*), respectively. Standard quality-control procedures [36] were performed with PLINK 1.9 [37], resulting in 6,910,431 SNPs in the *Training set* and 6,552,380 SNPs in the *Validation set* (Supplementary Material). Only those individuals with valid BMI measures were included (Supplementary Table S1). Finally, a PCA was performed on the resulting individuals and the top 10 PCs were kept for further analysis.

### Pleiotropy analyses with GWAS data

GWAS summary statistics on BMI were obtained from Pulit et al. [38], which comprised association analyses of a total of 806,834 European individuals. For SCZ, we obtained GWAS data on 67,390 patients and 94,015 controls, with 80% being of European ancestry [1] (Supplementary Table S2). GWAS summary statistics were referenced to 9,546,816 SNPs generated from the 1,000 Genomes Project (1KGP). SNPs that were nonbiallelic, without rsIDs, duplicated, or with strand-ambiguous alleles were removed. We also filtered out SNPs with INFO scores < 0.9, those mapping to the extended major histocompatibility complex (MHC, chr6: 25,119,106–33,854,733), SNPs located on chromosomes X, Y, and mitochondria, and SNPs with sample sizes 5 standard deviations away from the mean. Finally, a common set of 1,949,409 SNPs were kept in the two datasets. ORs and betas were transformed into *z*-scores.

We used *pleioFDR* [25] to identify genetic loci jointly associated with the two phenotypes, as previously described [27] (Supplementary Material). SNPs jointly associated with BMI and SCZ (conj. FDR < 0.05) were mapped to genes with ANNOVAR [39]. Genelists were submitted to KOBAS-I [40] to check for enrichment in biological categories and diseases; and to the GENE2FUNC option implemented in FUMA to check for tissue enrichment [41]. All protein-coding genes were used as background and the Benjamini–Hochberg (BH) method was used for false discovery rate (FDR) correction.

To obtain the widest representation of the SNPs in the pleiotropic loci between SCZ and BMI, SNPs that were in *r* ^2^ > 0.1, distance < 250 kb, and MAF > 0.001 with each independent SNPs at conj. FDR < 0.05 were recovered with PLINK v1.9 from the CEU population of the 1 kG project. After this process, all the obtained SNPs were classified as pleiotropic SNPs. Nonpleiotropic SNPs were defined as all the SNPs in the original BMI GWAS that were not considered in any pleiotropic locus.

### PRS estimation

PRSice 2.3.1.e was used to implement a pipeline in PRS creation [42], using a two-step procedure as previously developed [43]. This avoids sample overlap between the *Training* and *Validation sets and* prevents test statistic inflation. First, PRSs were calibrated in the *Training set* using the GWAS on BMI [38] as base data. Scores were calculated for multiple *p*-threshold cutoffs (from 5e-08 to 1 with increments of 5e-05) using *r* ^2^ = 0.1, 250-kb window, SNPs with INFO > 0.9, and excluding the MHC. Then, the optimal *p*-value (*p*_optimal_) threshold was determined as the one with the highest prediction for each phenotype. Sex, age, and the 10 first PCs were included as covariates (when evaluating ∆BMI, BMI_0_ was also included as a covariate). Second, SNPs below each obtained *p*_optimal_ threshold were identified and carried forward for PRS computation in individuals of the *Validation set.*

### Statistical analyses

First, Mann–Whitney and Kruskal–Wallis tests were used to assess differences in ∆BMI at 12 months (∆BMI_12_) based on clinical characteristics, use of antidepressants, and the AP drug described. Chi-squared tests were performed to evaluate differences between the *Training* and *Validation* sets.

To elucidate whether the PRS_BMI_ were associated with the BMI in our dataset we carried out multiple linear regressions in the *Validation set.* We designed our analysis in three phases, first, we constructed linear models with the observed BMI_12_, ∆BMI_3_, and ∆BMI_12_ as the dependent variables, which combined only clinical and demographic variables. These models were referred to as *Clinical models.* They included as independent variables the 10 first PCs (to adjust for population stratification), sex, age, AP dose and the type of AP drug (at the corresponding time point), the diagnosis, tobacco smoking, and cannabis use. When the ∆BMI were evaluated, BMI_0_ was also included as a covariable. We also performed *Clinical models* with the whole dataset to determine each variable’s contribution in the BMI. Second, to examine the predictive ability of including genetic factors in the *Validation set*, the PRS_BMI_ values obtained were subsequently incorporated into the previous models. Thus, we produced a series of absolute risk models with the observed BMI (or ∆BMI) as the dependent variable, and the covariates in the *Clinical model* plus the PRS_BMI_ as independent variables, named *PRS models.* Third, we divided the genome-wide significant SNPs (*p* < 5e-08) from the BMI GWAS into pleiotropic and nonpleiotropic, using information from the *pleioFDR* analysis, to build additional *PRS models* which independently included PRS derived from pleiotropic (PRS_pleio_) and nonpleiotropic (PRS_nonpleio_) loci. In these cases, only SNPs that belonged to each category were included. Finally, we used the ANOVA() function to compare whether *PRS models* were significantly different from the *Clinical models.* All the analyses were performed in R 3.6.0 [44].

Since all the BMI measures were highly interrelated, a Bonferroni correction was considered too restrictive for the linear regression models. Instead, the alpha level was corrected by estimating the effective number of tests [45]. In our study, the resulting significance threshold was set at *p* = 0.027.

## Results

### Characteristics of the whole dataset

A total of 381 patients were included in the entire dataset. Among them, 188 (49.3%) were diagnosed with schizophrenia, 115 (30.2%) with schizophreniform disorder, 43 (11.3%) with brief psychotic disorder, 28 (7.4%) with not otherwise specified psychosis, 5 (1.3%) with schizoaffective disorder, and 2 (0.5%) with delusional disorder (Table 1). At the end of the study, 89.2% of the patients had all BMI measures. None of the participants was initially treated with clozapine, which is indicated in treatment-resistant SCZ [46] (Table 2). A total of 354 patients (92.9%) were AP-naïve. The other 27 patients (7.1%) had been treated with AP prior to their inclusion in the study, although during a short period of time (mean 10.4 days, SD: 11.2 days; median 5 days; range: 1–42 days).

In the dataset, the mean ∆BMI_3_ and ∆BMI_12_ were 6.4 and 13.9%, respectively (Supplementary Figure S1). Most of the individuals displayed a positive ∆BMI_12_ (*n* = 312, 91.8%), while 25 individuals (7.4%) showed a negative ∆BMI_12_, and 3 individuals (<0.1%) had the same BMI_0_ and BMI_12_. In total, 108 patients in the *Training set* (48.2%) and 94 in the *Validation set* (59.9%) remained with the same AP drug during the study (Supplementary Figure S2). Both ∆BMI_3_ and ∆BMI_12_ were negatively correlated with BMI_0_ (*p* = 5.56e-10 and *p* = 7.07e-07) and positively correlated with each other (Supplementary Figure S3). In the whole sample, all APs were associated, on average, with a positive ∆BMI (Supplementary Figure S4). There were differences in ∆BMI_12_ between AP drugs among the individuals that did not switch AP drug during the study period (*p* = 1.9e-03), being olanzapine associated with a higher ∆BMI_12_. There were also differences in tobacco smokers versus nonsmokers (*p* = 0.01) and in cannabis users versus nonusers (*p* = 2.5e-03). However, ∆BMI_12_ was not associated with diabetes, metabolic syndrome, alcohol use, being AP *drug naïve*, or not having been prescribed antidepressants and mood stabilizers at baseline (Supplementary Figure S5). To reduce the number of variables in the models, only significant factors were incorporated into the subsequent prediction models.

### Pleiotropy between SCZ and BMI

**Figure 1.**
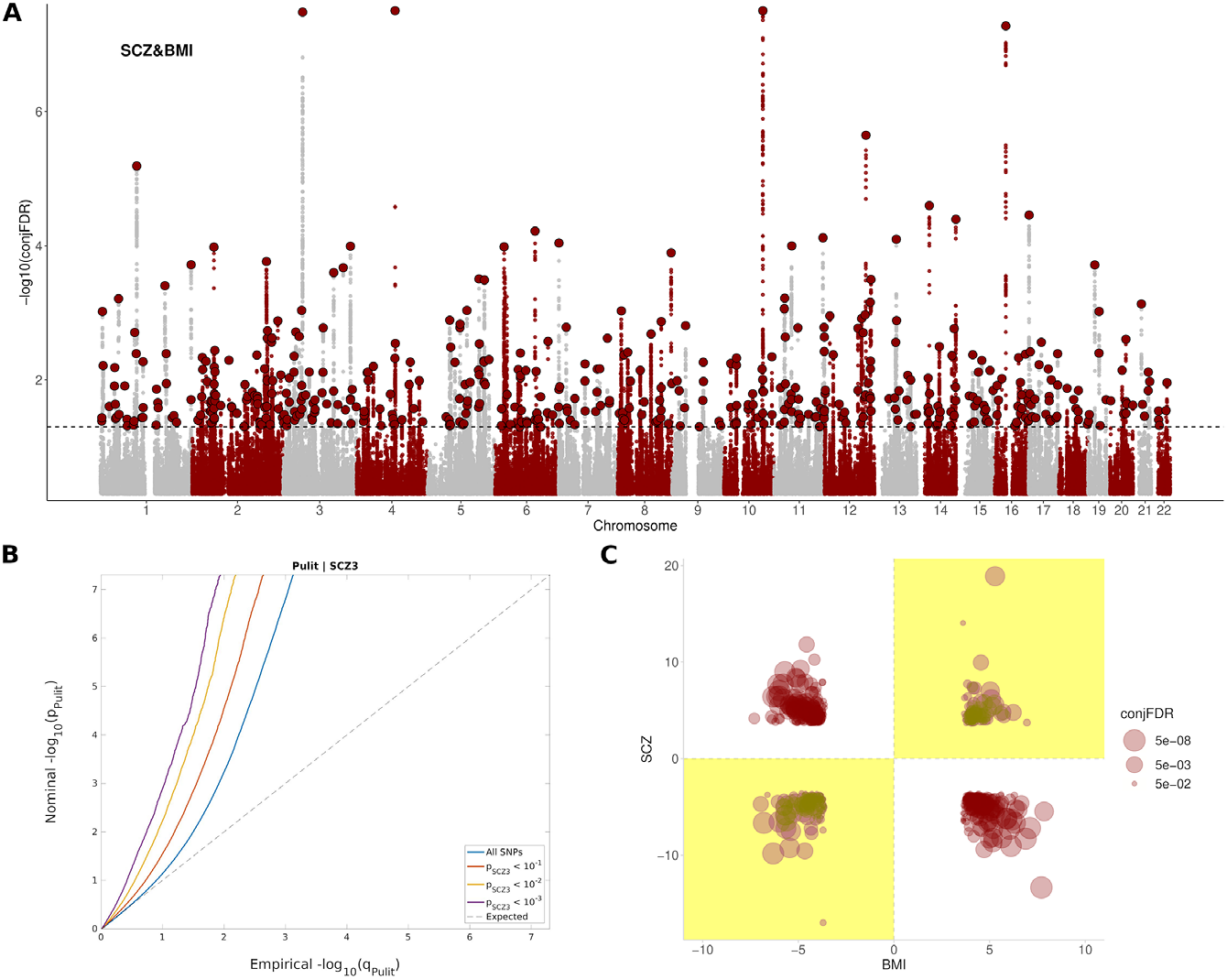
Pleiotropic variants between SCZ and BMI. (A) Manhattan plot showing independent (*r* ^2^ < 0.1) loci associated with both SCZ and BMI, as defined by conjunction false discovery rates (conj. FDR) after excluding SNPs in the MHC region. The dashed black line represents the conj. FDR threshold of 0.05. (B) Conditional Q–Q plots of nominal versus empirical (−log10) *p*-values (corrected for inflation) of BMI as a function of significance with SCZ, at the level of *p* < 10^−1^ (red line), *p* < 10^−2^ (yellow line), and *p* < 10^−3^ (purple line), respectively. The blue line indicates the standard enrichment of BMI including all SNPs, irrespective of their association with the secondary trait. The gray dashed line indicates the null distribution of *p*-values. (C) Pleiotropy plot for independent SNPs with conj. FDR < 0.05 (*n* = 486) between SCZ and BMI. The conj. FDR values and the direction of the effects (*z*-scores) of the minor alleles are plotted for BMI (*x*-axis) against SCZ (*y*-axis). Graph regions whose effects are consistent with a positive correlation between the two traits are shaded in yellow. (C) The ratio between discordant and concordant pleiotropic variants across different conj. FDR thresholds.

A total of 486 independent SNPs belonging to 449 near-independent genomic loci (*r* ^2^ < 0.1) were identified as being jointly associated with SCZ and BMI at conj. FDR < 0.05 (Figure 1A,B and Supplementary Table S3). Among them, 169 independent SNPs (35%) showed concordant relationships, where the alleles that confer risk for SCZ, also increase BMI. In addition, 317 independent SNPs (65%) showed discordant links, in which the allele that increased SCZ-risk, had a BMI reduction effect (Figure 1C). This represented an approximate twofold ratio between discordant (antagonistic) and concordant (agonistic) pleiotropic variants that were conserved across significance thresholds (Supplementary Figure S6). Neither of the two groups of variants differed in their effect sizes in SCZ (*p* = 0.45) or BMI (*p* = 0.17), and nor in their minor allele frequency (*p* = 0.72).

The set of SNPs shared between SCZ and BMI included 9,262 SNPs corresponding to 889 genes in total, enriched in Alcoholism (FDR = 5,15e-15), DNA methylation (FDR = 4,99e-14), DNA damage (FDR = 7,52e-13), cAMP signaling pathway (FDR = 2,45e-09), axon guidance (FDR = 1,06e-07), dopaminergic synapse (FDR = 2,99e-05), insulin resistance (FDR = 6,57e-03), and nervous system diseases (FDR = 7,58e-05), among others (Supplementary Table S4 and Supplementary Figure S7). These genes were differentially regulated in the brain; and were especially up-regulated in the frontal cortex, anterior cingulate cortex, putamen, amygdala, nucleus accumbens, and hippocampus (Supplementary Figure S8). A complete list of pathways enriched in both agonistic and antagonistic loci can be found in Supplementary Tables S5 and S6.

### Risk models for BMI and ∆BMI

**Figure 2.**
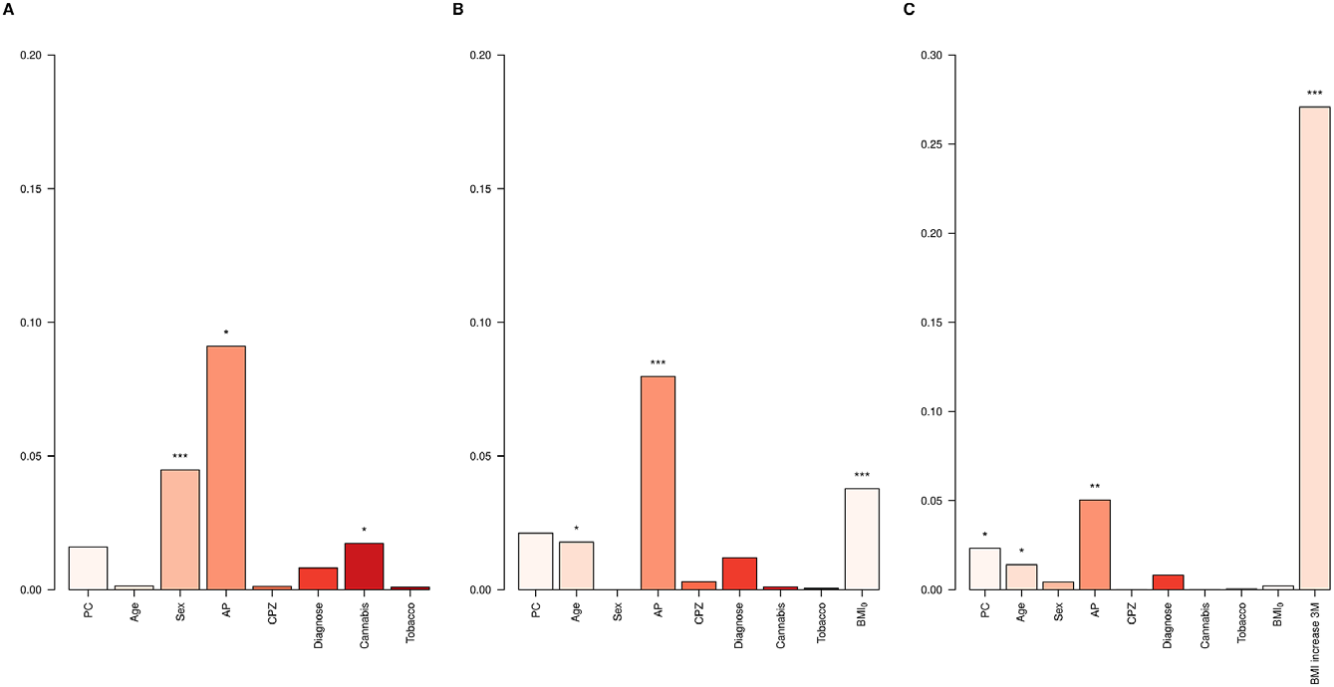
∆BMI variance explained in the whole dataset. Bar plots showing the variance explained by each covariate in the *Clinical models* for (A) BMI_12_, (B) ∆BMI_12_, and (C) ∆BMI_12_ including ∆BMI_3_ in the model. AP, antipsychotic drug; CPZ, equivalent doses of chlorpromazine. *p-value < 0.05; **p-value < 0.01; ***p–value < 0.001.

First, the *Clinical models* for BMI_12_ and ∆BMI_12_ were evaluated in the entire dataset. Variables associated with a higher BMI_12_ were male sex (*p* = 7.4e-05), being on haloperidol (*p* = 1.6e-04), olanzapine (*p* = 6.1e-03), risperidone (*p* = 6.8e-03), and quetiapine treatment (*p* = 0.03), and not consuming cannabis (*p* = 0.01, Supplementary Table S7). Among them, the major contributors to BMI_12_ were sex (4.5%), AP treatment (9.1%) and not being a cannabis user (1.7%, Figure 2A). On the other hand, variables associated with higher ∆BMI_12_ were reduced age (*p* = 0.01), low BMI_0_ (*p* = 2.4e-04), and AP treatment with paliperidone (*p* = 8.2e-03) and clozapine (*p* = 8.8e-03) at the 3-month follow-up (Supplementary Table S7). BMI_0_ contributed 3.8% to the total variance of ∆BMI_12_, AP treatment 8%, and age 1.8% (Figure 2B). When ∆BMI_3_ was included, it was the major contributor, accounting for 27.1% of ∆BMI_12_ variance (Figure 2C).

**Figure 3.**
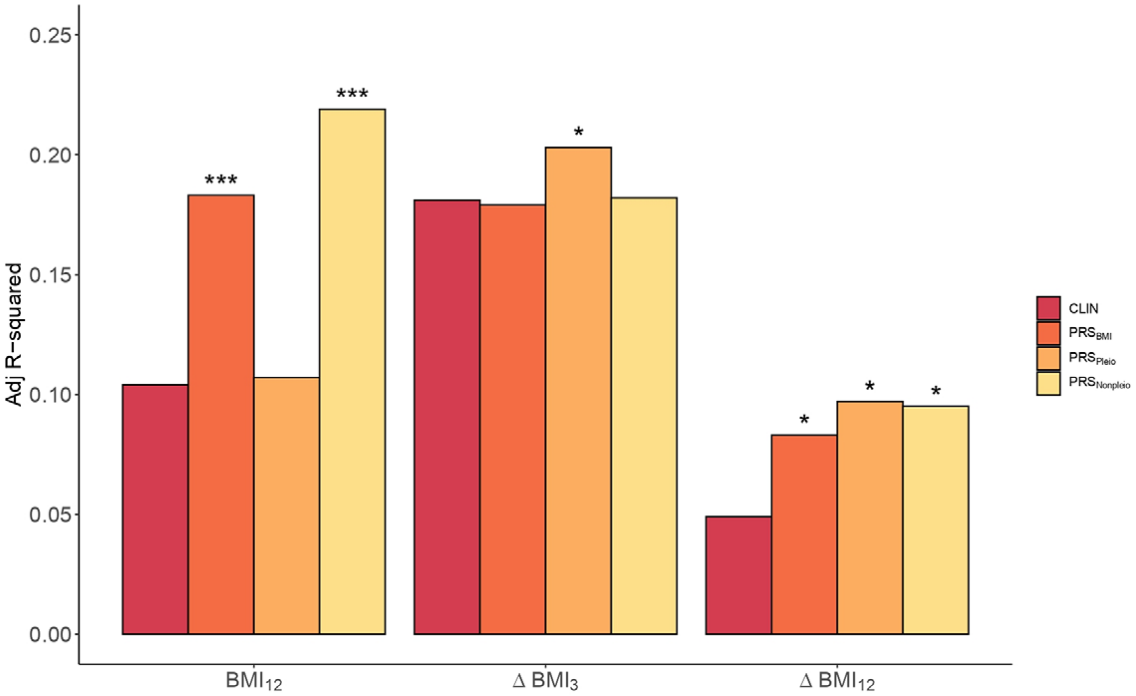
*Clinical* versus *PRS models* in BMI. Barplots showing the Adj. *R* ^2^ in BMI by the *Clinical model* (CLIN) and the *PRS models* computed using all SNPs from BMI GWAS (PRS_BMI_), pleiotropic SNPs (PRS_Pleio_), and nonpleiotropic SNPs (PRS_Nonpleio_) in the *Validation dataset* (*n* = 157). The barplot shows predictions of BMI_12_, ΔBMI_3_, and ΔBMI_12_ in the *x*-axis. Covariates included in the *Clinical model* were the first 10 PC, age, sex, AP drug prescribed, chlorpromazine equivalent doses, diagnose, tobacco smoking, and cannabis use. Each *PRS model* was compared to the performance of the corresponding *Clinical model.* Asterisks represent significantly improved models compared to the *Clinical models* (ANOVA). *p-value < 0.05; ***p–value < 0.001.

To assess the performance of adding PRS in predicting BMI, the whole dataset was split into a *Training set* and a *Validation set.* The *p*_optimal_ was brought forward to calculate PRS_BMI_ in the *Validation set* and the scores were included into the *PRS models* for comparison against the *Clinical models.* For BMI_12_ the *p*_optimal_ was established at 0.15 (Supplementary Table S8). The inclusion of the PRS_BMI_ improved the *Clinical model* for BMI_12_ by 76% (*p* = 7.7e-04, Adj. *R* ^2^_CLIN_ = 0.10, Adj. *R* ^2^_PRS_ = 0.18, Figure 3 and Supplementary Table S9).

Next, we studied the inclusion of PRS_BMI_ for predicting ∆BMI_3_ and ∆BMI_12_. The *p*_optimal_ thresholds in the *Training set* were estimated at 2.2e-03 and 0.13, respectively (Supplementary Table S8). The percentage increase in the *PRS models* was 69.4% in ∆BMI_12_ (*p* = 0.02, Adj. *R* ^2^_CLIN_ = 0.05; Adj. *R* ^2^_PRS_ = 0.08), but not significant in ∆BMI_3_ (*p* = 0.39, Adj. *R* ^2^_CLIN_ = 0.18; Adj. *R* ^2^_PRS_ = 0.18, Supplementary Table S10).

Given the large extent of genetic pleiotropy between SCZ and BMI, we investigated whether these variants played a specific role in determining BMI and ∆BMI in our cohort. We obtained two PRSs based on pleiotropic (PRS_pleio_) and nonpleiotropic SNPs (PRS_nonpleio_, Supplementary Figure S9). The PRS_pleio_ was not a significant predictor in determining absolute BMI_12_ (*p* = 0.26), as confirmed by the comparison of the *PRS model* including the PRS_pleio_ (Adj. *R* ^2^_Pleio_ = 0.11) compared to the *Clinical model* (Adj. *R* ^2^_CLIN_ = 0.10, Figure 3). Instead, the PRS_nonpleio_ showed an improvement in BMI_12_ higher than that of PRS_BMI_: 110.6% (Adj. *R* ^2^_Nonpleio_ = 0.22, *p* = 5.3e-05). In contrast, including PRS_pleio_ improved the ∆BMI_12_ model by 98% (*p* = 0.01, Adj. *R* ^2^_CLIN_ = 0.05, Adj. *R* ^2^_Pleio_ = 0.10, Figure 3), as well as the ∆BMI_3_ model by 12.2% (*p* = 0.04, Adj. *R* ^2^_CLIN_ = 0.18, Adj. *R* ^2^_Pleio_ = 0.20), indicating the role of the pleiotropic variants in weight increase. PRS_nonpleio_ was not relevant for predicting ∆BMI_3_ (*p* = 0.28); however, it significantly improved ∆BMI_12_ by 93.9% (*p* = 0.01, Adj. *R* ^2^_Nonpleio_ = 0.10). Finally, we classified the pleiotropic variants into agonistic and antagonistic to construct both agonistic PRSs (PRS_ago_) and antagonistic PRSs (PRS_antag_). In predicting both ∆BMI_3_ and ∆BMI_12_, the PRS_antag_ outperformed the PRS_ago_ (Supplementary Figure S10).

## Discussion

Understanding the genetic vulnerability associated with increased BMI can be used to predict the risk of ∆BMI prior to treatment initiation so that personalized risk-based treatments can be implemented. In this study, we demonstrate for the first time, the replicable effects of a PRS_BMI_ in predicting ∆BMI in FEP, with a prominent role of variants shared between SCZ and BMI.

In our study, BMI_0_ was inversely associated with ∆BMI_3_ and ∆BMI_12_, indicating that individuals with lower BMI_0_ were more likely to have higher ∆BMI during the first year of treatment [47], which is in line with a previous study on an extended FEP cohort [48]. In the analyses of patients who did not switch AP, greater BMI gain was observed in patients treated with atypical AP, in accordance with previous studies [49, 50]. However, the effects varied greatly within medications, and interactions with underlying individual characteristics and genetic factors may be relevant [51].

Previous studies have proposed that the weight gain in psychosis is associated with the altered expression of genes related to both obesity and BMI [52], which suggests that there is a genetic overlap between these two medical conditions. Here, we confirm and extend with further data the shared genetic architecture between BMI and SCZ, involving pathways such as alcoholism (alcohol use disorder), DNA damage, DNA methylation, insulin resistance, and dopaminergic and glutamatergic synapses [28], which are promising mechanisms for understanding weight gain in FEP. Alcohol intake can be a contributing factor to weight gain, probably by effects on central neurotransmitter systems to increase appetite [53], however, it has to be highlighted that alcohol use disorder was among the exclusion criteria for entering the PAFIP program. Specific DNA methylation signatures have been widely described to play a role in obesity and weight loss in humans [54–56]. There is also evidence pointing to the relevance of the glutamatergic system as a promising strategy to treat obesity [57]. In addition, weight gain associated with clozapine may be linked to antagonism of the histaminergic H1 receptors, increasing the risk of insulin resistance and type 2 diabetes [58]. The pleiotropic variants between the two traits belong to genes that are up-regulated in the frontal and anterior cingulate cortices, the putamen, amygdala, nucleus accumbens, and hippocampus, which suggests that these areas play a relevant role in weight increase associated with SCZ. For instance, dopamine has been considered to be the target responsible for the efficacy of AP and also to be involved in feeding behavior, and the accumbens is considered to be the brain area with an increased release of dopamine [59]. In fact, nucleus accumbens microstructure can be used to predict weight increase in children [60]. Similarly, hippocampus size has been discovered to be a predictor for change in BMI in FEP [61].

A recent study in FEP found no association between psychopathological PRS with metabolic progression, including BMI [62]. However, in the present study, we demonstrate the role of underlying genetics in both BMI and ∆BMI in FEP patients. Including PRS_BMI_ ostensibly improved the prediction of BMI at 12 months of treatment. This is not surprising, as the role of genetics in BMI in the general population is already known [31]; however, this is the first time it is also validated in FEP individuals under AP treatment. Notably, we report for the first time, that the inclusion of PRS_BMI_ also improved the prediction of mid-term ∆BMI, which may have important consequences in identifying FEP patients at high risk for weight gain. Strikingly, including PRS_pleio_ improved both risk models of ∆BMI_12_ and ∆BMI_3_, although containing a much lower number of SNPs. Remarkably, ∆BMI_12_ was strongly predicted by ∆BMI_3_ (explaining almost 30% of its variance). This result emphasizes that clinicians should focus on the early weeks of treatment to prevent long-term weight gain [63], also reinforcing the benefits of including pleiotropy information for early detection of BMI increase in FEP. In contrast, the genetic architecture of BMI not shared with SCZ (PRS_nopleio_) played a pivotal role in controlling BMI. Our results are in line with recent data showing that incorporating pleiotropic information improves prediction by capturing biological mechanisms shared between traits [64, 65]. Moreover, opening the door to applying it to the study of comorbidity between *a priori* independent traits. Our results also indicate that antagonistic variants (those that have opposite directions between BMI and SCZ) play a greater role in ∆BMI. However, it could be simply a matter of statistical power as more SNPs were recovered in this category.

This study has one main strength: it is based on independent longitudinal and prospective cohorts of well-characterized drug-naïve FEP patients in which different types of AP medication were considered. However, our work has some limitations; first, we could not control for well-known factors that contribute to BMI changes, such as diet and physical activity [66, 67]. Secondly, the interpretation of the results could also be skewed by the fact that FEP patients often switch AP treatment during follow-up, and are sometimes treated with a secondary AP, which have a possible impact on BMI [68]. It is noteworthy that the two datasets do not have the same distribution of AP treatments. Although this may imply a bias when the results between AP drugs are compared, it also reinforces the role of genetics in ∆BMI given its replicability in sets of individuals with different treatments. Also, the fact that GWAS of SCZ contained a 20% non-European sample may have slightly biased the calculation of pleiotropic regions and, in turn, the pleiotropic PRS. Finally, the predictive power we obtain with a small population (*N* < 200) is relatively low (Adj. *R* ^2^ ~ 0.1–0.2), but is fully comparable to that of other studies with much larger cohorts [32, 69–71]. Further research with larger cohorts and including populations of non-European ancestry is needed to better understand this relationship and to develop effective interventions to address the issue of weight gain in people with FEP.

In summary, our findings highlight that genetics is an important factor in determining the BMI trajectory in patients with FEP, paving the way for its inclusion in the clinical routine in order to identify individuals at higher risk, and to optimize individualized prevention programs to improve patients’ quality of life. In turn, our results lay the groundwork for addressing the prediction of comorbid trajectories in other diseases using a similar approach.

## Data Availability

The datasets used in the current study are available from the authors upon request.

## Supplementary material

For supplementary material accompanying this paper visit http://doi.org/10.1192/j.eurpsy.2023.9. click here to view supplementary material

## References

[1] Trubetskoy V, Pardiñas AF, Qi T, Panagiotaropoulou G, Awasthi S, Bigdeli TB, et al. Mapping genomic loci implicates genes and synaptic biology in schizophrenia. Nature. 2022;604(7906):502–8. 35396580 10.1038/s41586-022-04434-5 PMC9392466

[2] Krebs MD, Themudo GE, Benros ME, Mors O, Børglum AD, Hougaard D, et al. Associations between patterns in comorbid diagnostic trajectories of individuals with schizophrenia and etiological factors. Nat Commun. 2021;12(1):6617. 34785645 10.1038/s41467-021-26903-7 PMC8595374

[3] Mitchell AJ, Vancampfort D, Sweers K, van Winkel R, Yu W, De Hert M. Prevalence of metabolic syndrome and metabolic abnormalities in schizophrenia and related disorders - a systematic review and meta-analysis. Schizophr Bull. 2013;39(2):306–18. 22207632 10.1093/schbul/sbr148 PMC3576174

[4] Kurdyak P, Mallia E, de Oliveira C, Carvalho AF, Kozloff N, Zaheer J, et al. Mortality after the first diagnosis of schizophrenia-spectrum disorders: a population-based retrospective cohort study. Schizophr Bull. 2021;47(3):864–74. 33459778 10.1093/schbul/sbaa180 PMC8084423

[5] Rødevand L, Steen NE, Elvsåshagen T, Quintana DS, Reponen EJ, Mørch RH, et al. Cardiovascular risk remains high in schizophrenia with modest improvements in bipolar disorder during past decade. Acta Psychiatr Scand. 2019;139(4):348–60. 30697685 10.1111/acps.13008

[6] Vázquez-Bourgon J, Ibáñez Alario M, Mayoral-van Son J, Gómez Revuelta M, Ayesa Arriola R, Juncal Ruiz M, et al. A 3-year prospective study on the metabolic effect of aripiprazole, quetiapine and ziprasidone: a pragmatic clinical trial in first episode psychosis patients. Eur Neuropsychopharmacol. 2020;39:46–55. 32891516 10.1016/j.euroneuro.2020.08.009

[7] Pillinger T, McCutcheon RA, Vano L, Mizuno Y, Arumuham A, Hindley G, et al. Comparative effects of 18 antipsychotics on metabolic function in patients with schizophrenia, predictors of metabolic dysregulation, and association with psychopathology: a systematic review and network meta-analysis. Lancet Psychiatry. 2020;7(1):64–77. 31860457 10.1016/S2215-0366(19)30416-X PMC7029416

[8] Allison DB, Mackell JA, McDonnell DD. The impact of weight gain on quality of life among persons with schizophrenia. Psychiatr Serv. 2003;54(4):565–7. 12663847 10.1176/appi.ps.54.4.565

[9] Dayabandara M, Hanwella R, Ratnatunga S, Seneviratne S, Suraweera C, de Silva V. Antipsychotic-associated weight gain: management strategies and impact on treatment adherence. NDT. 2017;13:2231–41. 10.2147/NDT.S113099 PMC5574691 28883731

[10] McWhinney S, Kolenic M, Franke K, Fialova M, Knytl P, Matejka M, et al. Obesity as a risk factor for accelerated brain ageing in first-episode psychosis—a longitudinal study. Schizophrenia Bulletin. 2021;47:1772–81. 34080013 10.1093/schbul/sbab064 PMC8530396

[11] Wilson PWF, D’Agostino RB, Sullivan L, Parise H, Kannel WB. Overweight and obesity as determinants of cardiovascular risk: the Framingham experience. Arch Intern Med. 2002;162(16):1867–72. 12196085 10.1001/archinte.162.16.1867

[12] Correll CU, Detraux J, De Lepeleire J, De Hert M. Effects of antipsychotics, antidepressants and mood stabilizers on risk for physical diseases in people with schizophrenia, depression and bipolar disorder. World Psychiatry. 2015;14(2):119–36. 26043321 10.1002/wps.20204 PMC4471960

[13] Pérez-Iglesias R, Martínez-García O, Pardo-Garcia G, Amado JA, Garcia-Unzueta MT, Tabares-Seisdedos R, et al. Course of weight gain and metabolic abnormalities in first treated episode of psychosis: the first year is a critical period for development of cardiovascular risk factors. Int J Neuropsychopharmacol. 2014;17(1):41–51. 24103107 10.1017/S1461145713001053

[14] Bioque M, García-Portilla MP, García-Rizo C, Cabrera B, Lobo A, González-Pinto A, et al. Evolution of metabolic risk factors over a two-year period in a cohort of first episodes of psychosis. Schizophr Res. 2018;193:188–96. 28663026 10.1016/j.schres.2017.06.032

[15] Gebhardt S, Haberhausen M, Heinzel-Gutenbrunner M, Gebhardt N, Remschmidt H, Krieg JC, et al. Antipsychotic-induced body weight gain: predictors and a systematic categorization of the long-term weight course. J Psychiatr Res. 2009;43(6):620–6. 19110264 10.1016/j.jpsychires.2008.11.001

[16] Vázquez-Bourgon J, Mayoral-van Son J, Gómez-Revuelta M, Juncal-Ruiz M, Ortiz-García de la Foz V, Tordesillas-Gutiérrez D, et al. Treatment discontinuation impact on long-term (10-year) weight gain and lipid metabolism in first-episode psychosis: Results from the PAFIP-10 cohort. Int J Neuropsychopharmacol. 2021;24(1):1–7. 32840607 10.1093/ijnp/pyaa066 PMC7816683

[17] Raben AT, Marshe VS, Chintoh A, Gorbovskaya I, Müller DJ, Hahn MK. The complex relationship between antipsychotic-induced weight gain and therapeutic benefits: A systematic review and implications for treatment. Front Neurosci. 2017;11:741. 29403343 10.3389/fnins.2017.00741 PMC5786866

[18] Theisen FM, Gebhardt S, Haberhausen M, Heinzel-Gutenbrunner M, Wehmeier PM, Krieg JC, et al. Clozapine-induced weight gain: a study in monozygotic twins and same-sex sib pairs. Psychiatr Genet. 2005;15(4):285–9. 16314759 10.1097/00041444-200512000-00011

[19] Zhang JP, Lencz T, Zhang RX, Nitta M, Maayan L, John M, et al. Pharmacogenetic associations of antipsychotic drug-related weight gain: a systematic review and meta-analysis. Schizophr Bull. 2016;42(6):1418–37. 27217270 10.1093/schbul/sbw058 PMC5049532

[20] Müller DJ, Kennedy JL. Genetics of antipsychotic treatment emergent weight gain in schizophrenia. Pharmacogenomics. 2006;7(6):863–87. 16981847 10.2217/14622416.7.6.863

[21] Gebhardt S, Theisen FM, Haberhausen M, Heinzel-Gutenbrunner M, Wehmeier PM, Krieg JC, et al. Body weight gain induced by atypical antipsychotics: an extension of the monocygotic twin and sib pair study: Twin/sib study on antipsychotic-induced weight gain. J Clin Pharm Ther. 2010;35(2):207–11. 20456740 10.1111/j.1365-2710.2009.01084.x

[22] Ter Hark SE, Jamain S, Schijven D, Lin BD, Bakker MK, Boland-Auge A, et al. A new genetic locus for antipsychotic-induced weight gain: a genome-wide study of first-episode psychosis patients using amisulpride (from the OPTiMiSE cohort). J Psychopharmacol. 2020;34(5):524–31. 32126890 10.1177/0269881120907972 PMC7222287

[23] Maciukiewicz M, Tiwari AK, Zai CC, Gorbovskaya I, Laughlin CP, Nurmi EL, et al. Genome-wide association study on antipsychotic-induced weight gain in Europeans and African-Americans. Schizophr Res. 2019;212:204–12. 31447353 10.1016/j.schres.2019.07.022

[24] Lee PH, Anttila V, Won H, Feng YCA, Rosenthal J, Zhu Z, et al. Genomic relationships, novel loci, and pleiotropic mechanisms across eight psychiatric disorders. Cell. 2019;179(7):1469–82.e11. 31835028 10.1016/j.cell.2019.11.020 PMC7077032

[25] Andreassen OA, Djurovic S, Thompson WK, Schork AJ, Kendler KS, O’Donovan MC, et al. Improved detection of common variants associated with schizophrenia by leveraging pleiotropy with cardiovascular-disease risk factors. Am J Hum Genet. 2013;92(2):197–209. 23375658 10.1016/j.ajhg.2013.01.001 PMC3567279

[26] Smeland OB, Bahrami S, Frei O, Shadrin A, O’Connell K, Savage J, et al. Genome-wide analysis reveals extensive genetic overlap between schizophrenia, bipolar disorder, and intelligence. Mol Psychiatry. 2020;25(4):844–53. 30610197 10.1038/s41380-018-0332-x PMC6609490

[27] Muntané G, Farré X, Bosch E, Martorell L, Navarro A, Vilella E. The shared genetic architecture of schizophrenia, bipolar disorder and lifespan. Hum Genet. 2021;140:441–55. 32772156 10.1007/s00439-020-02213-8

[28] Bahrami S, Steen NE, Shadrin A, O’Connell K, Frei O, Bettella F, et al. Shared genetic loci between body mass index and major psychiatric disorders: a genome-wide association study. JAMA Psychiat. 2020;77(5):503–12. 10.1001/jamapsychiatry.2019.4188 PMC6990967 31913414

[29] Duncan LE, Shen H, Ballon JS, Hardy KV, Noordsy DL, Levinson DF. Genetic correlation profile of schizophrenia mirrors epidemiological results and suggests link between polygenic and rare variant (22q11.2) cases of schizophrenia. Schizophr Bull. 2018;44(6):1350–61. 29294133 10.1093/schbul/sbx174 PMC6192473

[30] Ikeda M, Tanaka S, Saito T, Ozaki N, Kamatani Y, Iwata N. Re-evaluating classical body type theories: genetic correlation between psychiatric disorders and body mass index. Psychol Med. 2018;48(10):1745–8. 29651975 10.1017/S0033291718000685 PMC6088781

[31] Khera AV, Chaffin M, Wade KH, Zahid S, Brancale J, Xia R, et al. Polygenic prediction of weight and obesity trajectories from birth to adulthood. Cell. 2019;177(3):587–96.e9. 31002795 10.1016/j.cell.2019.03.028 PMC6661115

[32] Krapohl E, Patel H, Newhouse S, Curtis CJ, von Stumm S, Dale PS, et al. Multi-polygenic score approach to trait prediction. Mol Psychiatry. 2018;23(5):1368–74. 28785111 10.1038/mp.2017.163 PMC5681246

[33] Pelayo-Terán JM, Pérez-Iglesias R, Ramírez-Bonilla M, González-Blanch C, Martínez-García O, Pardo-García G, et al. Epidemiological factors associated with treated incidence of first-episode non-affective psychosis in Cantabria: insights from the clinical programme on early phases of psychosis. Early Interv Psychiatry. 2008;2(3):178–87. 21352151 10.1111/j.1751-7893.2008.00074.x

[34] Gómez-Revuelta M, Pelayo-Terán JM, Juncal-Ruiz M, Vázquez-Bourgon J, Suárez-Pinilla P, Romero-Jiménez R, et al. Antipsychotic treatment effectiveness in first episode of psychosis: PAFIP 3-year follow-up randomized clinical trials comparing haloperidol, olanzapine, risperidone, aripiprazole, quetiapine, and ziprasidone. Int J Neuropsychopharmacol. 2020;23(4):217–29. 31974576 10.1093/ijnp/pyaa004 PMC7177160

[35] Gardner DM, Murphy AL, O’Donnell H, Centorrino F, Baldessarini RJ. International consensus study of antipsychotic dosing. AJP. 2010;167(6):686–93. 10.1176/appi.ajp.2009.09060802 20360319

[36] Marees AT, de Kluiver H, Stringer S, Vorspan F, Curis E, Marie-Claire C, et al. A tutorial on conducting genome-wide association studies: quality control and statistical analysis. Int J Methods Psychiatr Res. 2018;27(2):e1608. 29484742 10.1002/mpr.1608 PMC6001694

[37] Purcell S, Neale B, Todd-Brown K, Thomas L, Ferreira MAR, Bender D, et al. PLINK: a tool set for whole-genome association and population-based linkage analyses. Am J Hum Genet. 2007;81(3):559–75. 17701901 10.1086/519795 PMC1950838

[38] Pulit SL, Stoneman C, Morris AP, Wood AR, Glastonbury CA, Tyrrell J, et al. Meta-analysis of genome-wide association studies for body fat distribution in 694 649 individuals of European ancestry. Hum Mol Genet. 2019;28(1):166–74. 30239722 10.1093/hmg/ddy327 PMC6298238

[39] Wang K, Li M, Hakonarson H. ANNOVAR: Functional annotation of genetic variants from high-throughput sequencing data. Nucleic Acids Res. 2010;38(16):e164. 20601685 10.1093/nar/gkq603 PMC2938201

[40] Bu D, Luo H, Huo P, Wang Z, Zhang S, He Z, et al. KOBAS-i: intelligent prioritization and exploratory visualization of biological functions for gene enrichment analysis. Nucleic Acids Res. 2021;49(W1):W317–25. 34086934 10.1093/nar/gkab447 PMC8265193

[41] Watanabe K, Taskesen E, van Bochoven A, Posthuma D. Functional mapping and annotation of genetic associations with FUMA. Nat Commun. 2017;8(1):1826. 29184056 10.1038/s41467-017-01261-5 PMC5705698

[42] Choi SW, O’Reilly PF. PRSice-2: polygenic risk score software for biobank-scale data. Gigascience. 2019;8(7):giz082. 31307061 10.1093/gigascience/giz082 PMC6629542

[43] Khera AV, Chaffin M, Aragam KG, Haas ME, Roselli C, Choi SH, et al. Genome-wide polygenic scores for common diseases identify individuals with risk equivalent to monogenic mutations. Nat Genet. 2018;50(9):1219–24. 30104762 10.1038/s41588-018-0183-z PMC6128408

[44] R Core Team. R: a language and environment for statistical computing. Vienna, Austria: R Foundation for Statistical Computing, https://www.R-project.org/; 2021.

[45] Derringer J. A simple correction for non-independent tests [Internet]. PsyArXiv [citat 5 juliol 2022], https://osf.io/f2tyw; 2018.

[46] Kane JM, Agid O, Baldwin ML, Howes O, Lindenmayer JP, Marder S, et al. Clinical guidance on the identification and management of treatment-resistant schizophrenia. J Clin Psychiatry. 2019;80(2):18com12123. 10.4088/JCP.18com12123 30840788

[47] Manu P, Dima L, Shulman M, Vancampfort D, De Hert M, Correll CU. Weight gain and obesity in schizophrenia: epidemiology, pathobiology, and management. Acta Psychiatr Scand. 2015;132(2):97–108. 26016380 10.1111/acps.12445

[48] Canal-Rivero M, Ruiz-Veguilla M, Labad J, Ayesa-Arriola R, Vázquez-Bourgon J, Mayoral-van Son J, et al. Predictors of weight acquisition induced by antipsychotic treatment and its relationship with age in a sample of first episode non-affective psychosis patients: a three-year follow-up study. Schizophr Res. 2020;222:462–4. 32600780 10.1016/j.schres.2020.06.011

[49] Hugenholtz GW, Heerdink ER, Meijer WE, Stolker JJ, Egberts AC, Nolen WA. Reasons for switching between antipsychotics in daily clinical practice. Pharmacopsychiatry. 2005;38(3):122–4. 15902582 10.1055/s-2005-864122

[50] Lieberman JA, Tollefson G, Tohen M, Green AI, Gur RE, Kahn R, et al. Comparative efficacy and safety of atypical and conventional antipsychotic drugs in first-episode psychosis: a randomized, double-blind trial of olanzapine versus haloperidol. Am J Psychiatry. 2003;160(8):1396–404. 12900300 10.1176/appi.ajp.160.8.1396

[51] Lett TAP, Wallace TJM, Chowdhury NI, Tiwari AK, Kennedy JL, Müller DJ. Pharmacogenetics of antipsychotic-induced weight gain: review and clinical implications. Mol Psychiatry. 2012;17(3):242–66. 21894153 10.1038/mp.2011.109

[52] Crespo-Facorro B, Prieto C, Sainz J. Altered gene expression in antipsychotic-induced weight gain. NPJ Schizophr. 2019;5(1):7. 30971689 10.1038/s41537-019-0075-y PMC6458173

[53] Traversy G, Chaput JP. Alcohol consumption and obesity: an update. Curr Obes Rep. 2015;4(1):122–30. 25741455 10.1007/s13679-014-0129-4 PMC4338356

[54] Dick KJ, Nelson CP, Tsaprouni L, Sandling JK, Aïssi D, Wahl S, et al. DNA methylation and body-mass index: a genome-wide analysis. The Lancet. 2014;383(9933):1990–8. 10.1016/S0140-6736(13)62674-4 24630777

[55] Samblas M, Milagro FI, Martínez A. DNA methylation markers in obesity, metabolic syndrome, and weight loss. Epigenetics. 2019;14(5):421–44. 30915894 10.1080/15592294.2019.1595297 PMC6557553

[56] Tobi EW, Slieker RC, Luijk R, Dekkers KF, Stein AD, Xu KM, et al. DNA methylation as a mediator of the association between prenatal adversity and risk factors for metabolic disease in adulthood. Sci Adv. 2018;4(1):eaao4364. 29399631 10.1126/sciadv.aao4364 PMC5792223

[57] Oliveira TPD, Gonçalves BDC, Oliveira BS, de Oliveira ACP, Reis HJ, Ferreira CN, et al. Negative modulation of the metabotropic glutamate receptor type 5 as a potential therapeutic strategy in obesity and binge-like eating behavior. Front Neurosci. 2021;15:631311. 33642987 10.3389/fnins.2021.631311 PMC7902877

[58] Guest PC. Insulin resistance in schizophrenia. In: Guest PC, editor. Reviews on biomarker studies of metabolic and metabolism-related disorders. Cham: Springer International Publishing; 2019, p. 1–16 (Advances in Experimental Medicine and Biology, vol. 1134), http://link.springer.com/10.1007/978-3-030-12668-1_1. 10.1007/978-3-030-12668-1_1 30919329

[59] Panariello F, De Luca V, de Bartolomeis A. Weight gain, schizophrenia and antipsychotics: new findings from animal model and pharmacogenomic studies. Schizophr Res Treatment. 2011;2011:459284. 22988505 10.1155/2011/459284 PMC3440684

[60] Rapuano KM, Laurent JS, Hagler DJ, Hatton SN, Thompson WK, Jernigan TL, et al. Nucleus accumbens cytoarchitecture predicts weight gain in children. Proc Natl Acad Sci U S A. 2020;117(43):26977–84. 33046629 10.1073/pnas.2007918117 PMC7604478

[61] Luckhoff HK, du Plessis S, Kilian S, Asmal L, Scheffler F, Phahladira L, et al. Hippocampal subfield volumes and change in body mass over 12 months of treatment in first-episode schizophrenia spectrum disorders. Psychiatry Res Neuroimaging. 2020;300:111084. 32388386 10.1016/j.pscychresns.2020.111084

[62] Segura ÀG, Martínez-Pinteño A, Gassó P, Rodríguez N, Bioque M, Cuesta MJ, et al. Metabolic polygenic risk scores effect on antipsychotic-induced metabolic dysregulation: a longitudinal study in a first episode psychosis cohort. Schizophr Res. 2022;244:101–10. 35659654 10.1016/j.schres.2022.05.021

[63] Kinon BJ, Kaiser CJ, Ahmed S, Rotelli MD, Kollack-Walker S. Association between early and rapid weight gain and change in weight over one year of olanzapine therapy in patients with schizophrenia and related disorders. J Clin Psychopharmacol. 2005;25(3):255–8. 15876905 10.1097/01.jcp.0000161501.65890.22

[64] Loika Y, Irincheeva I, Culminskaya I, Nazarian A, Kulminski AM. Polygenic risk scores: pleiotropy and the effect of environment. Geroscience. 2020;42(6):1635–47. 32488673 10.1007/s11357-020-00203-2 PMC7732954

[65] van der Meer D, Shadrin AA, O’Connell K, Bettella F, Djurovic S, Wolfers T, et al. Boosting schizophrenia genetics by utilizing genetic overlap with brain morphology. Biol Psychiatry. 2022;92(4):291–8. 35164939 10.1016/j.biopsych.2021.12.007 PMC12012303

[66] Firth J, Stubbs B, Teasdale SB, Ward PB, Veronese N, Shivappa N, et al. Diet as a hot topic in psychiatry: a population-scale study of nutritional intake and inflammatory potential in severe mental illness. World Psychiatry. 2018;17(3):365–7. 30192082 10.1002/wps.20571 PMC6127755

[67] Vancampfort D, Firth J, Schuch FB, Rosenbaum S, Mugisha J, Hallgren M, et al. Sedentary behavior and physical activity levels in people with schizophrenia, bipolar disorder and major depressive disorder: a global systematic review and meta-analysis. World Psychiatry. 2017;16(3):308–15. 28941119 10.1002/wps.20458 PMC5608847

[68] Correll CU, Rummel-Kluge C, Corves C, Kane JM, Leucht S. Antipsychotic combinations vs monotherapy in schizophrenia: a meta-analysis of randomized controlled trials. Schizophr Bull. 2009;35(2):443–57. 18417466 10.1093/schbul/sbn018 PMC2659301

[69] Hüls A, Wright MN, Bogl LH, Kaprio J, Lissner L, Molnár D, et al. Polygenic risk for obesity and its interaction with lifestyle and sociodemographic factors in European children and adolescents. Int J Obes. 2021;45(6):1321–30. 10.1038/s41366-021-00795-5 PMC8159747 33753884

[70] de Toro-Martín J, Guénard F, Bouchard C, Tremblay A, Pérusse L, Vohl MC. The challenge of stratifying obesity: attempts in the Quebec family study. Front Genet. 2019;10:994. 31649740 10.3389/fgene.2019.00994 PMC6796792

[71] Murthy VL, Xia R, Baldridge AS, Carnethon MR, Sidney S, Bouchard C, et al. Polygenic risk, fitness, and obesity in the coronary artery risk development in young adults (CARDIA) study. JAMA Cardiol. 2020;5(3):40–8. 31913407 10.1001/jamacardio.2019.5220 PMC6990863

